# Longitudinal Rehabilitation of Binocular Function in Adolescent Intermittent Exotropia After Successful Corrective Surgery

**DOI:** 10.3389/fnins.2021.685376

**Published:** 2021-07-05

**Authors:** Tingting Peng, Meiping Xu, Fuhao Zheng, Junxiao Zhang, Shuang Chen, Jiangtao Lou, Chunxiao Wang, Yuwen Wang, Xinping Yu

**Affiliations:** ^1^The Eye Hospital, School of Ophthalmology and Optometry, Wenzhou Medical University, Wenzhou, China; ^2^State Key Laboratory of Ophthalmology, Zhongshan Ophthalmic Center, Sun Yat-sen University, Guangzhou, China

**Keywords:** intermittent exotropia, strabismus surgery, binocular function, rehabilitation, ocular alignment

## Abstract

**Purpose:**

To study the longitudinal rehabilitation of binocular visual function in adolescent intermittent exotropia (IXT) after successful surgery and compare the results with those of a normal population. The role of binocular function in ocular alignment stability was also evaluated postoperatively.

**Methods:**

In this prospective study, 30 adolescents with IXT successfully corrected after 1 month were followed for 12 months, and 30 children with normal vision were enrolled as controls. Stereopsis, the fusional vergence amplitude, sensory fusion, and accommodative flexibility were measured to assess binocular function at baseline and 6 and 12 months postoperatively. The controls were tested once when they were enrolled in the study.

**Results:**

The deviation was −32.00 ± 8.60 prism diopters (PD) at distance fixation and −36.0 ± 9.10 PD at near fixation preoperatively with an average correction of 28.53 ± 3.79 PD and 30.67 ± 1.34 PD at 1 month postoperatively. Distance stereoacuity and near stereoacuity improved from 1 to 12 months postoperatively (*p* = 0.025 and *p* = 0.041, respectively). Compared with the controls, the fusional convergence reserve at distance (*p* = 0.025) and near (*p* = 0.033) fixations and fusion reserve ratio at distance (*p* = 0.000) and near (*p* = 0.000) fixations remained subnormal, whereas sensory fusion (*p* = 0.237), distance stereopsis (*p* = 0.120), and the fusional divergence amplitude at a distance (*p* = 0.168) were normal. However, no significant correlations were found between binocular functions at 1 month postoperatively and the postoperative drift.

**Conclusion:**

Binocular function significantly improved from before to after successful corrective surgery and continued to improve from 1 to 12 months postoperatively in adolescents with IXT. No significant correlations were found between binocular functions at 1 month postoperatively and ocular alignment stability.

## Introduction

Intermittent exotropia (IXT) is the most common form of exotropia in children and adolescents. The prevalence of IXT is approximately 1% in Western countries (Hutchinson, 2001) and approximately 3% in teenagers in China (Pan et al., 2016). IXT is characterized by intermittent fusion when the eyes experience proper ocular alignment in the early stage of strabismus, leading to the development of binocularity and stereopsis; patients with IXT possess the potential to regain normal binocular functions after treatment (Donahue, 2007). Surgical treatment is critical for IXT (Jie et al., 2010; Yu et al., 2016) in children and adolescents. Binocular functions (e.g., stereopsis, sensory fusion, and motor fusion) improve after surgery (O’Neal et al., 1995; Yildirim et al., 1999; Wu et al., 2006; Adams et al., 2008; Sharma et al., 2008; Feng X. et al., 2015; Pineles et al., 2015; Wu Y. et al., 2020). Feng X. et al. (2015) found that stereopsis and sensory fusion improved from 2 to 6 weeks postoperatively, whereas binocular fusion and alignment were not evaluated over longer periods. Distance stereoacuity improved in individuals with IXT at 6 weeks (Adams et al., 2008) and 1 year after successful surgery (Yildirim et al., 1999; Wu et al., 2006). Sharma et al. (2008) found an increase in fusional vergence and both near and distance stereoacuities 6 months after surgery in individuals with IXT. However, these studies performed only one follow-up after surgery. Longitudinal rehabilitation in binocular function after successful surgery as well as their associations with ocular alignment drift were not evaluated. The longitudinal recovery of binocular function in IXT patients after successful surgery should be evaluated extensively.

The high recurrence rate after surgery reduces the benefits of corrective surgery in IXT patients. The specific factors for recurrence or drift after surgery in IXT have not yet been established. However, factors, such as the surgical procedure, age at surgery, angle of deviation, and preoperative binocular functions, have been studied previously (Pineles et al., 2010; Lee et al., 2018; Mohan and Sharma, 2020; Repka et al., 2020). Postoperative binocular function was not related to postoperative ocular alignment stability in our retrospective study (Wu Y. et al., 2020). An adequate understanding of the recovery of binocular function after successful surgery may help identify the factors associated with the recurrence of IXT after surgery.

Based on these findings, we designed a prospective study to evaluate the longitudinal rehabilitation of binocular visual function (e.g., sensory fusion, stereoacuity, fusional vergence amplitude, amplitude of accommodation, accommodative flexibility) after successful surgery to compare the results with those of a normal population and to determine whether binocular function is involved in the stability of ocular alignment postoperatively.

## Subjects and Methods

This prospective case–control study was approved by the Ethics Committee of the Eye Hospital of Wenzhou Medical University and was performed in accordance with the Declaration of Helsinki.

### Subjects

Thirty patients aged 7–17 years with IXT successfully corrected for 1 month after surgery and 30 asymptomatic age- and sex-matched controls were recruited from the Eye Hospital of Wenzhou Medical University from July 2019 to September 2019. Only patients with basic IXT (Donahue et al., 2019) preoperatively were included in the study. The definition of successful motor alignment is orthotropia, X(T) ≤ 10 prism diopters (PD) and E(T) ≤ 5 PD in the primary position at distance and near fixations (Kim and Choi, 2016; Donahue et al., 2019). Patients were excluded if any of the following conditions were encountered: diplopia at 1 month after surgery, vertical deviation ≥ 5 PD, dissociated vertical deviation (DVD), oblique muscle dysfunction, an A or V pattern, eye movement restricted in one direction, a congenital cranial nerve abnormality, a history of extraocular muscle surgery or botulinum toxin injection treatment, amblyopia (≥2 lines interocular difference on Snellen’s vision chart), anisometropia (a spherical or cylindrical difference ≥ 2.0 diopters), or a history of binocular vision therapy pre- or postoperatively.

### Data Collection

The following data were recorded for each patient: name, sex, age, age at the time of surgery, best-corrected visual acuity (BCVA), eye movements (EOM), preoperative and postoperative angle of deviation and binocular functions, including sensory (sensory fusion, stereoacuity) and motor function (fusional vergency amplitude, amplitude of accommodation, and accommodative flexibility).

The angle of deviation was measured using the prism and alternate cover test (PACT) at 33 cm for near and 6 m for distance fixations. The deviation of tropia was measured using the simultaneous prism and cover test (SPCT) at 33 cm for near fixation and 6 m for distance fixation (Donahue et al., 2019).

Sensory fusion was tested using the Worth 4-dot test at distance and near fixations. Patients who detected four dots were considered to have fusion, those who saw five dots were considered diplopic, and those who saw two or three dots were considered to have suppression. Subjects who saw 2 or 3 dots at distance fixation and 4 dots at near fixation were defined as having peripheral fusion (Yildirim et al., 1999; Feng X. et al., 2015).

Near stereoacuity was assessed using TNO stereopsis tests at 40 cm (Laméris Ootech B.V., Nieuwegein, Netherlands), ranging from 15 to 480 s of arc (arcsec) (Wu Y. et al., 2020). Distance stereoacuity was assessed using the Distance Randot Stereotest (DRS, American Stereo Optical Company) at 3 m, ranging from 63 to 400 arcsec (Wang et al., 2010). Stereoacuity was recorded as “nil” if the patient could not pass the largest disparity (Hatt et al., 2008). The stereopsis examination was performed before any other examination that required binocular fusion to be broken.

The fusional vergence amplitude was measured using a horizontal prism bar, and an accommodative target was used first at distance fixation (3 m) and then at near fixation (1/3 m) (Sharma et al., 2008; Hatt et al., 2011) with base-in (BI) for negative vergence and base-out (BO) for positive vergence. Negative fusional vergence was measured before positive vergence to prevent bias caused by the prismatic demand of positive fusional vergence. To determine the fusional convergence break point (convergence reserve), the magnitude of prism was gradually increased from 1 PD until diplopia appeared with no subsequent recovery of motor fusion or one eye drifted outwards when control was lost. If the patient could still perform fusion up to the maximum prism volume of 45^△^, the break point was recorded as 45^△^ for statistical analysis (Hatt et al., 2011). If a patient could not perform fusion, both the break point and recovery point were recorded as 0. The total fusional convergence amplitude (Hatt et al., 2011) was the sum of the individual deviation angle and convergence reserve. The fusion reserve ratio (Hatt et al., 2011) was calculated as the fusional convergence reserve divided by the angle of deviation measured using the PACT (e.g., fusional convergence reserve = 20; angle of deviation = 10; fusion reserve ratio = 2).

For the near point of convergence (NPC), the patient looked at an accommodative target located 40 cm away, and the examiner gradually moved the target toward the patient’s eyes until the patient reported that the target had become two targets. The distance between the break point and parallel point of the patient’s lateral canthus was measured.

The amplitude of accommodation (AMP) was measured using the negative lens method, and the right eye’s data were used for analysis.

Binocular accommodative flexibility (BAF) was measured by reading the “E” visual acuity chart at 40 cm in sequence with a ± 2.00 D reverse lens within 1 min (García et al., 2000; Wick et al., 2002). Positive and negative counts comprise one cycle. The measurement starts with the positive lens, and the number of cycles that occur within 1 min is recorded.

All the tests were performed after appropriate refractive correction. Each of these tests was performed at 1, 6, and 12 months postoperatively. Two patients were lost to follow-up at 6 months after surgery. The controls were tested once when they were enrolled in the study.

### Statistical Analysis

Postoperative drift was defined as the change in ocular alignment from the 1-month follow-up to the final follow-up. The Friedman test was used to compare the angle of deviation, stereoacuity, and fusional vergence amplitude (1, 6, and 12 months). The Wilcoxon signed-rank test was used to compare rehabilitation between 1 and 12 months postoperatively, and the Mann–Whitney U test was used to compare IXT patients and controls. The sensory fusion status at each time point (1, 6, and 12 months) was evaluated using chi-squared test or Fisher’s exact test. Additionally, we evaluated the relationships between postoperative binocular functions and postoperative drift using Spearman’s correlation coefficient.

## Results

Thirty cases and 30 age- and sex-matched controls were included. A summary of the subjects’ demographics and clinical characteristics is provided in Table 1. The deviations at both distance and near fixations decreased significantly from preoperatively to 1 month postoperatively (*p* < 0.001), sensory fusion improved (*p* = 0.009), and distance stereoacuity improved (*p* = 0.026); however, near stereoacuity did not recover significantly (*p* = 0.657).

**TABLE 1:** Demographics and clinical characteristics of the patients with IXT and normal controls.

Characteristic	IXT patients				Normal controls
	Preoperatively	1 month postoperatively	6 months postoperatively	12 months postoperatively	
Sex: female, male	–	11, 19	11, 17	11, 19	10, 20
Age (years)	–	10.87 ± 2.40	–	–	9.90 ± 2.06
SE of the right eye (D)	–	−2.20 ± 2.38	−2.11 ± 2.13	−2.48 ± 2.37	−0.97 ± 1.33
SE of the left eye (D)	–	−2.00 ± 2.39	−1.67 ± 2.05	−1.90 ± 2.77	−0.87 ± 1.20
Sensory fusion (fusion, peripheral fusion, no fusion)	15, 9, 6	26, 3, 1	27, 1, 0	27, 3, 0	30, 0, 0
Distance stereoacuity (log arcsec)	3.55 ± 0.77	3.18 ± 0.97	2.79 ± 0.88	2.77 ± 0.85	2.33 ± 0.63
Near stereoacuity (log arcsec)	2.24 ± 0.44	2.20 ± 0.34	2.06 ± 0.34	2.03 ± 0.35	1.98 ± 0.29
Distance deviation (PD)	-32.0 ± 8.60	−3.47 ± 4.81	−5.36 ± 6.63	−7.67 ± 7.70	−0.23 ± 0.57
Near deviation (PD)	−36.0 ± 9.10	−5.33 ± 7.76	−7.86 ± 8.29	−10.30 ± 9.87	−2.27 ± 3.04
Distance base-out	–	15.43 ± 13.78	15.50 ± 11.89	18.70 ± 15.14	23.70 ± 9.90
Distance base-in	–	6.14 ± 4.47	7.68 ± 5.02	10.23 ± 5.58	8.67 ± 2.37
Near base-out	–	21.43 ± 15.39	22.14 ± 11.14	24.67 ± 14.48	32.37 ± 9.29
Near base-in	–	9.86 ± 6.41	11.89 ± 6.93	17.07 ± 8.28	11.90 ± 3.97
NPC (cm)	–	4.00 ± 4.21	6.13 ± 4.61	4.13 ± 4.53	2.25 ± 2.88
Binocular accommodative flexibility (BAF)	–	9.67 ± 4.30	11.64 ± 3.97	12.38 ± 3.45	9.03 ± 3.34
AMP (D)	–	8.83 ± 3.26	8.33 ± 2.47	8.18 ± 2.16	7.37 ± 2.16

*D, diopters. PD, prism diopters.*

*The angle of deviation was measured using the PACT. Base-out and base-in were the break points. Two patients were lost to follow-up 6 months postoperatively. AMP, the right eye’s amplitude of accommodation.*

### Postoperative Drift

**FIGURE 1:**
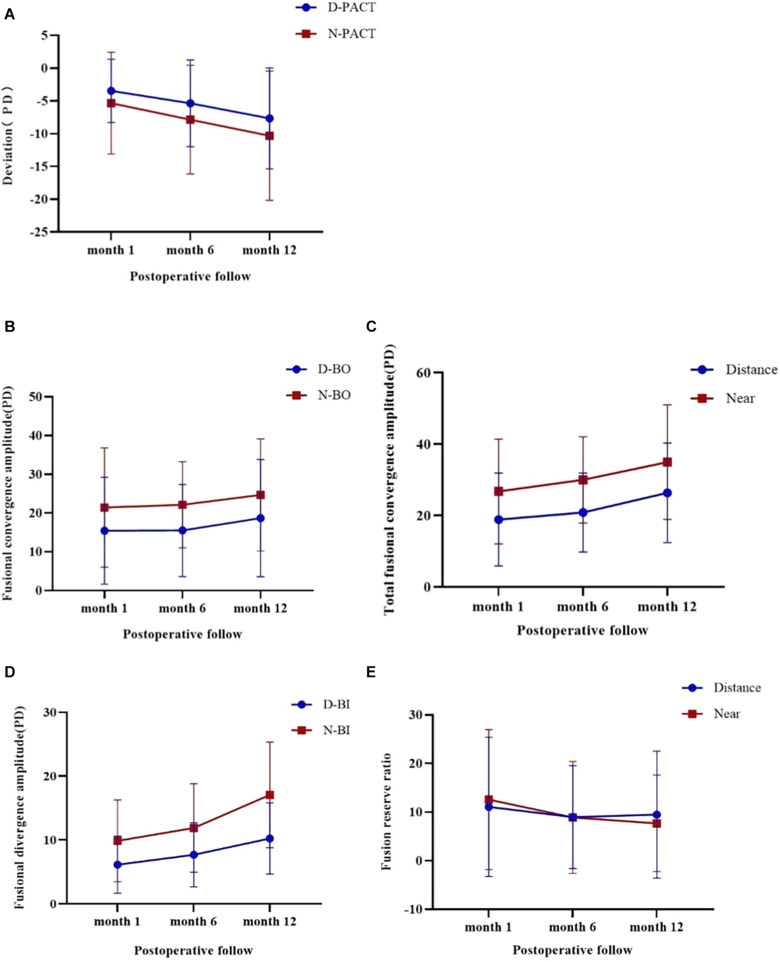
Changes in the eye position and fusional vergence amplitude during the three follow-ups after surgery and using the Friedman test. A negative value indicates an exodrift. **(A)** Shows that the eye position steadily drifts outwards for distance and near fixations (*p* < 0.001). **(B)** Shows that the BO break point leads to different recoveries in distance and near fixations (*p* = 0.045 and *p* = 0.000, respectively). **(C)** Shows that the total fusional convergence amplitude has different recoveries in distance and near fixations (*p* = 0.169 and *p* = 0.000, respectively). **(D)** Shows that the BI break point has different increases in distance and near fixations (*p* < 0.001). **(E)** Shows that the fusion reserve ratio worsens in distance and near fixations (*p* = 0.001 and *p* < 0.001, respectively).

Figure 1A shows that the eye position steadily drifted outwards. The mean exodrift at distance fixation was 1.53 ± 7.19 PD from postoperative month 1 to postoperative month 6 and 2.67 ± 9.38 PD from postoperative month 6 to postoperative month 12 (*p* = 0.338), and the mean exodrift values at near fixation were 2.00 ± 9.83 PD and 2.97 ± 11.62 PD, respectively (*p* = 0.459). No relationship was found between the preoperative deviation and postoperative drift at distance or near fixations [*r*s = 0.352 (*p* = 0.056) and *r*s = 0.048 (*p* = 0.802), respectively]. The greater was the amount of preoperative deviation, the greater was the postoperative drift at a distance. However, the difference was not statistically significant.

### Binocular Functions After Surgery

#### Sensory Fusion

No significant differences were found in sensory fusion between 1 and 12 months postoperatively (*p* = 0.601) or between the patient and control groups (*p* = 0.117 and *p* = 0.237, respectively).

#### Stereoacuity

The patients’ median value of distance stereoacuity improved from 3.18 (range, 1.8–4.0) at 1 month postoperatively to 2.77 (range, 1.8–4) at 12 months postoperatively (*p* = 0.025), and the values did not significantly differ from those of the controls (*p* = 0.120). The patients’ median value of near stereoacuity improved from 2.20 (range, 1.7–2.98) at 1 month postoperatively to 2.03 (range, 1.48–2.68) at 12 months postoperatively (*p* = 0.041), which was still poorer than that of the controls (*p* = 0.017). Distance stereoacuity before and after surgery (1 month and 12 months postoperatively) did not significantly differ [*r*s = 0.308 (*p* = 0.098) and *r*s = 0.210 (*p* = 0.266), respectively], whereas near stereoacuity was significantly different [*r*s = 0.492 (*p* = 0.006) and *r*s = 0.547 (*p* = 0.002), respectively]. These results demonstrated that the better was preoperative near stereoacuity, the better was the postoperative recovery.

#### Fusional Vergence Amplitude

The patients’ median fusional vergence amplitudes, total fusional convergence amplitudes, and fusion reserve ratio at distance or near fixation improved at the three follow-ups (Figure 1B–E). However, the number of distance and near BO break points in the IXT group at the final visit was still worse than that in the controls (*p* = 0.025 and *p* = 0.033, respectively). The number of BI break points in the IXT group at the final visit at distance fixation was similar to that of the controls, whereas the number at near fixation exceeded that of the controls (*p* = 0.168 and *p* = 0.003, respectively). The distance and near-total fusional convergence amplitudes in the IXT group were similar to those of the controls at the final visit (*p* = 0.619 and *p* = 0.935, respectively). The fusion reserve ratio at 12 months postoperatively differed significantly between the patient and control groups at both distance (*p* = 0.000) and near (*p* = 0.000) fixation.

#### Binocular Accommodative Flexibility

The patients’ median amount of BAF improved from 9.67 (range, 0–19) at 1 month postoperatively to 12.38 (range, 3–20) at 12 months postoperatively. The magnitude of improvement significantly differed at these three follow-ups (*p* = 0.021). The amount of BAF in the IXT group at 1 month postoperatively was not significantly different from that of the controls (*p* = 0.533) and was better than that in the controls at 12 months postoperatively (*p* = 0.001).

#### Accommodative Amplitude

The patients’ median accommodative amplitude in the right eye improved from 8.43 ± 3.81 at 1 month postoperatively to 8.64 ± 3.67 at 12 months postoperatively (*p* = 0.700), which was not significantly different from that of the controls (7.37 ± 2.16, *p* = 0.689 and *p* = 0.791, respectively).

### Relationship Between Postoperative Binocular Functions and Postoperative Drift

#### Relationship Between Stereoacuity and Postoperative Drift

**FIGURE 2:**
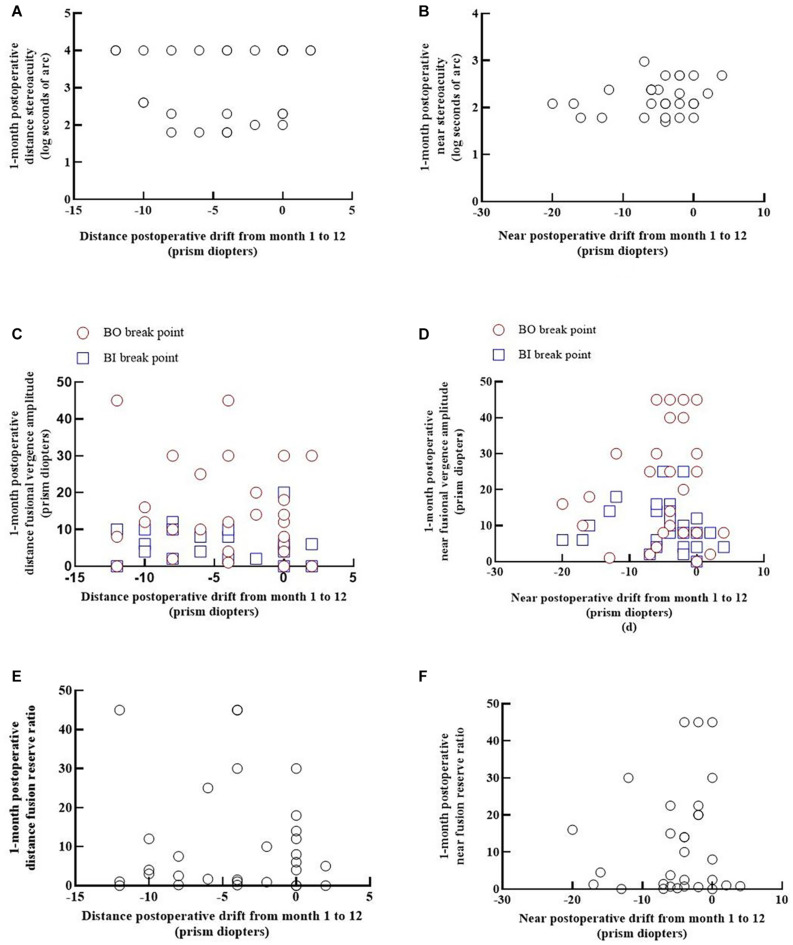
Relationships between postoperative binocular functions and postoperative drift at distance and near fixations. Each panel shows the data from 30 patients. The positive values indicate esodrift, and the negative values indicate exodrift. **(A,B)** Show the relationship between 1-month postoperative stereoacuity and the postoperative drift from months 1 to 12 for distance and near fixations [*r*s = 0.106 (*p* = 0.579) and *r*s = 0.143 (*p* = 0.450), respectively]. **(C,D)** Show the relationship between the convergence and divergence amplitude and postoperative drift at distance fixation (BO: *r*s = -0.115, *p* = 0.546; BI: *r*s = -0.336, *p* = 0.075) and at near fixation (BO: *r*s = 0.031, *p* = 0.870; BI: *r*s = -0.268, *p* = 0.159). **(E,F)** Show that the relationships between the 1-month postoperative fusion reserve ratio and postoperative drift from months 1 to 12 were not significant for distance or near fixation [*r*s = 0.003 (*p* = 0.987) and *r*s = 0.110 (*p* = 0.564), respectively].

Figures 2A,B show that the relationships between 1-month postoperative stereoacuity and postoperative drift for distance and near fixation were not significant [*r*s = 0.106 (*p* = 0.579) and *r*s = 0.143 (*p* = 0.450), respectively].

#### Relationship Between the Fusional Vergence Amplitude and Postoperative Drift

No significant correlations were found between the angle of postoperative drift and fusional vergence amplitudes at 1 month postoperatively for distance or near fixation in IXT patients (Figure 2).

### Classification of the IXT Group Into the Orthophoria/Heterophoria Group and Small Residual Manifest Angle Group

By combining the measurements of the Worth 4-dot test and simultaneous prism and cover test (SPCT) at 1 month after surgery, the IXT group was divided into the orthophoria/heterophoria group (26 cases) and small residual manifest angle group (three cases with suppression and one case with diplopia). All four patients in the residual manifest group recovered normal single vision and motor function at the 12-month postoperative visit. Ocular alignment became better controlled to heterophoria at 12 months postoperatively. The other three cases in the orthophoria/heterophoria group exhibited reduced binocular vision functions and exodrifted significantly during the follow-up at 12 months postoperatively; thus, the operation was not successful in these patients.

## Discussion

To our best knowledge, this is the first longitudinal study to evaluate the rehabilitation of binocular function in IXT after successful corrective surgery. Our results showed that most patients recovered binocular vision function after successful surgery, and the highest recovery rate was noted from postoperative months 1 to 6. This rate improved steadily over the next 6 months. Although the patients recovered binocular functions after strabismus surgery, some functions (i.e., near stereopsis, fusional convergence reserve, and the fusion reserve ratio) were still subnormal compared with those in controls. Few previous studies have evaluated the effects of postoperative binocular visual functions on the long-term stability of postoperative ocular alignment. Our study findings indicated no significant correlation between postoperative drift and postoperative binocular functions, suggesting that postoperative binocular functions may not be involved in the stability of postoperative ocular alignment.

Stereoacuity is typically considered an important indicator of the severity of IXT and is used to determine surgical indications, particularly for distance stereopsis (Stathacopoulos et al., 1993; von Noorden and Campos, 2002; Holmes et al., 2007). The degree of recovery of stereopsis in IXT was inconsistent. O’Neal and associates (O’Neal et al., 1995) demonstrated that even with excellent postoperative alignment, the distance stereoacuity, particularly in the random dot test, does not recover fully. Notably, the age range of the subjects in that study was large, ranging from 5 to 87 years. Sharma et al. (2008) found that the mean distance stereoacuity of patients at 6 months postoperatively became similar to that of normal subjects. Our study also confirmed that distance stereopsis improved to the same level as that of the normal controls at the 6- or 12-month follow-up. Adams et al. (2008) and Feng X. et al. (2015) found that near stereoacuity remained essentially unchanged following surgery. However, it should be noted that the subjects were assessed 6 weeks postoperatively and were not compared with controls. We found that near stereoacuity remained poorer in the patients at the last follow-up compared with that in the controls; however, it improved after surgery. Our results are consistent with those reported by Sharma et al. (2008), who found that even at 6 months postoperatively, the mean level of near stereopsis was still poor compared with that of control subjects.

The fusional vergence amplitude reflects a patient’s ability to “control” a latent or intermittent deviation in IXT (von Noorden and Campos, 2002; Adams et al., 2008) or a patient’s ability to maintain phoria in childhood X(T) (Wakayama et al., 2018). Kushner and Morton (1998) and Kushner (1999) found that exotropia becomes more pronounced when fusional convergence is suspended. Few studies have observed the postoperative fusional vergence amplitude and its longitudinal rehabilitation as well as its association with postoperative drift. The IXT group drifted outwards, and the exodeviation increased during the follow-up. As expected, the number of BI break points increased. We also found that the convergence reserve and total convergence amplitude improved in the follow-up period, but the values remained lower than those of the normal controls. This finding indicates that patients’ fusional convergence ability may improve after the successful correction of IXT. Sharma et al. (2008) found no significant differences in the fusional vergence amplitude between patients at 6 months postoperatively and normal subjects. However, adult subjects were included in the study, and fusion data were collected after the angle of deviation had been corrected using prisms. Furthermore, Liebermann et al. (2012) believe that common measurement methods may not reveal the true divergence amplitudes. Most IXT patients have normal fusional divergence at near fixation, and approximately half show decreased fusional divergence at distance fixation. Hatt et al. (2011) found that the fusion reserve ratio correlates well with exodeviation control and may be useful in grading the severity of IXT. According to Sheard’s criterion, the fusional convergence reserve should be twice the magnitude of the angle of deviation. Moreover, Yam et al. (2013) found that a fusional reserve ratio ≥ 2 was an indicator of good control in patients. However, the postoperative fusional reserve ratio showed a decreasing trend and was not significantly correlated with postoperative drift in this study. The decreasing trend may be related to the change in exodrift after the surgery.

Consistent with most previous studies, we found that sensory fusion improved from before to after the operation (O’Neal et al., 1995; Yildirim et al., 1999; Feng X. et al., 2015; Wu Y. et al., 2020) and remained stable during the follow-up period without a trend of deterioration.

BAF improved at the last follow-up and exceeded that of the normal group, and the accommodative amplitude remained stable during the follow-up period and slightly exceeded that of the normal controls. Ha and his colleagues (Ha et al., 2016) suggested that the rehabilitation of accommodative loads at near fixation increases more in IXT patients than in normal controls, whereas binocular fusion remains consistent. One possible explanation is that disparity is a major driver of accommodation, and extra accommodation is a consequence in IXT patients.

According to Yildirim and her colleagues (Yildirim et al., 1999), better distance stereoacuity and central fusion before surgery are frequently associated with better surgical success in X(T). Beneish and Flanders (1994) found that overcorrection of poor preoperative stereopsis resulted in significant improvements in the surgical success rate and suggested that individuals with poor preoperative stereopsis may have good long-term alignment stability postoperatively. In this study, we found no significant relationship between postoperative stereopsis and postoperative drift. Furthermore, postoperative fusional vergence functions—i.e., fusional convergence reserve, total convergence amplitude, and fusional divergence amplitude—were not associated with postoperative drift and could not predict drift in the follow-up period. Considering the findings of previous studies (Hatt et al., 2008) and our study, other factors may exist in addition to postoperative binocular functions that are involved in postoperative alignment. For example, the surgery method and age at surgery will also affect the long-term stability of ocular alignment postoperatively. In this study, the operations were performed by the same doctor, the surgical procedures were all unilateral recession–resection procedures, and the school-aged children were older than seven years. The factors that interfere with postoperative eye position stability are relatively limited. Previous studies (Feng L. et al., 2015; Zhou et al., 2017; Wu H. et al., 2020) have shown that visual perception disorders occur in IXT patients; sensory imbalance persisted in surgically corrected subjects although they had normal levels of stereopsis (Park and Kim, 2013), and the level of sensory imbalance was subnormal in the follow-up period (Leow et al., 2010).

In the present study, we chose postoperative month 1 as baseline to observe the rehabilitation in binocular visual functions with IXT after surgery and excluded any immediate responses and complications related to surgery (e.g., pain, edema, bleeding, and reactions to anaesthetics). Furthermore, to minimize the influence of other factors, we set strict inclusion criteria (i.e., amblyopia, anisometropia, and vertical deviation). Similarly, we excluded patients with overcorrection (>5 PD) at 1 month postoperatively. Overcorrection is correlated with long-term motor benefits but has potential negative effects on binocular function (Leow et al., 2010; Choi et al., 2011; Park and Kim, 2013).

Our study has limitations. First, the control score plays a vital role in assessing the patients’ ability to maintain phoria (Hatt and Gnanaraj, 2013; Wakayama et al., 2018). However, it is unsuitable for patients with IXT after surgery. Second, we included a limited number of patients, and the follow-up time was not sufficiently long. Binocular functions and eye position drift should be assessed in larger study populations and over longer periods. Binocular function measurements can be affected by the testing method—i.e., the fusional convergence reserve in IXT is greater when viewing a stereo target (Cooper, 1993) and reduced during intense light exposure (Campos and Cipolli, 1992). The testing conditions were uniform across all subjects in the present study; thus, there is likely no measurement bias in our results. Finally, we found a tendency of larger deviation before surgery with more drift after surgery, which would also affect the evaluation of the postoperative factors related to the postoperative alignment stability. A stricter definition of deviation before surgery and a larger sample would minimize the effect.

Binocular functions remained poorer in IXT patients at 1 month postoperatively than in the control group and continued to improve from 1 to 12 months. Some parameters of the binocular functions recovered to the same level as those in the normal controls, whereas some remained worse than those in the normal controls. No significant correlations were found between binocular functions after surgery and the stability of the alignment.

## Data Availability Statement

The original contributions presented in the study are included in the article/supplementary material, further inquiries can be directed to the corresponding author/s.

## Ethics Statement

The studies involving human participants were reviewed and approved by the Ethics Committee of the Eye Hospital of Wenzhou Medical University. Written informed consent to participate in this study was provided by the participants’ legal guardian/next of kin.

## Author Contributions

MX, FZ, JL, CW, and YW: technical assistance and guidance. JZ and SC: participating in the follow-up and contact patients to collect data. XY: research and academic guidance. All authors contributed to the article and approved the submitted version.

## Conflict of Interest

The authors declare that the research was conducted in the absence of any commercial or financial relationships that could be construed as a potential conflict of interest.
